# Genomic signatures of barley breeding for environmental adaptation to the new continents

**DOI:** 10.1111/pbi.14077

**Published:** 2023-07-27

**Authors:** Haifei Hu, Penghao Wang, Tefera Tolera Angessa, Xiao‐Qi Zhang, Kenneth J. Chalmers, Gaofeng Zhou, Camilla Beate Hill, Yong Jia, Craig Simpson, John Fuller, Alka Saxena, Hadi Al Shamaileh, Munir Iqbal, Brett Chapman, Parwinder Kaur, Olga Dudchenko, Erez Lieberman Aiden, Gabriel Keeble‐Gagnere, Sharon Westcott, David Leah, Josquin F. Tibbits, Robbie Waugh, Peter Langridge, Rajeev Varshney, Tianhua He, Chengdao Li

**Affiliations:** ^1^ Western Crop Genetics Alliance, Centre for Crop & Food Innovation, Food Futures Institute, College of Science, Health, Engineering and Education Murdoch University Western Australia Murdoch Australia; ^2^ Rice Research Institute & Guangdong Key Laboratory of New Technology in Rice Breeding Guangdong Academy of Agricultural Sciences Guangzhou China; ^3^ School of Agriculture, Food and Wine University of Adelaide South Australia Glen Osmond Australia; ^4^ The James Hutton Institute Dundee UK; ^5^ Genomics WA Harry Perkins Institute of Medical Research and Telethon Kids Institute, University of Western Australia Western Australia Nedlands Australia; ^6^ School of Agriculture & Environment (SAgE) the University of Western Australia Western Australia Perth Australia; ^7^ The Center for Genome Architecture, Department of Molecular and Human Genetics Baylor College of Medicine Texas Houston USA; ^8^ Center for Theoretical Biological Physics Rice University Texas Houston USA; ^9^ Shanghai Institute for Advanced Immunochemical Studies ShanghaiTech Pudong China; ^10^ Broad Institute of MIT and Harvard Massachusetts Cambridge USA; ^11^ Agriculture Victoria, Department of Jobs Precincts and Regions, Agribio, La Trobe University Victoria Bundoora Australia; ^12^ Department of Primary Industries and Regional Development Agriculture and Food Western Australia South Perth Australia; ^13^ Seed Force Victoria Shepparton Australia; ^14^ Centre for Crop & Food Innovation, State Agricultural Biotechnology Centre, Food Futures Institute Murdoch University Western Australia Perth Australia

**Keywords:** long‐read genome sequencing, crop breeding, environmental adaptation, solar radiation, genomic imprint, structural variation

Crops expanding from their centres of domestication towards a wide range of agroclimatic regions has led to significant phenotypic and genetic divergence between cultivated forms. Since its domestication in the Fertile Crescent about 10 000 years ago, barley accompanied the spread of agriculture into Europe during the 5th and 6th millennia BC. It was subsequently introduced to North America and Australia by European settlers in the 17th and 18th centuries. The Australian growing season is effectively determined by the soil moisture availability, which is different from that in many European and North American countries where barley is grown over the summer half of the year with frequent rainfall events. Breeding activities are expected to have shaped the barley genomes and selected genes for adaptation to the relevant agroclimatic conditions. Elucidating the genetic basis for adaptation to contrasting agroclimatic conditions will advance our understanding of crop adaptation and guide breeders in selecting varieties for future changing environments.

In this study, we sequenced and de novo assembled the genomes of two early barley varieties bred out in Australia, namely “Clipper”, and “Stirling” (Figure S1A, Methods S1). The assembly length of the Clipper and Stirling genomes are 4.28 Gb and 4.26 Gb with a contig N50 of 39.4 Mb and 36.9 Mb, respectively, (Table S1). *In‐situ* Hi‐C sequencing anchored 97% of sequences to seven chromosomes in both assemblies (Figure S1B,C). The whole‐genome shotgun sequence of 56 barley cultivars from Australia, Europe, and North America was first mapped to the Clipper reference genome to investigate the modern barley cultivars' phylogenetic relationships and population structures (Methods S2). Australian and North American barley show diverse genetic differentiation patterns (Figure S2, Table S2‐S6). The various genetic differentiation patterns across chromosomes may reflect the breeding selection targeting different genomic regions in Australia and North America.

Barley breeding in Australia centred on selecting varieties with fast development, that is, early flowering, to escape terminal heat during the maturation stage (He *et al*., 2022). We examined gene Presence/Absence variants (PAVs) between European, Australian, and North American barley. We found that selecting early flowering and photoperiod‐sensitivity in Australia has enriched phenology gene alleles with specific PAVs (Methods S3). Seventy genes in Australian barley show a significant change in the presence frequency compared with European barley (Table S7), with 17 genes in the flowering pathways, including genes involved in photoperiod and circadian clock (*HvCK2a* and *HvCO16*), vernalisation (*HvCBF10A*), and meristem response and development (*HvSOC1, HvBM5, HvBM7*) (Figure S2E).

We further compared the genomes of seven barley cultivars (i.e., Clipper and Stirling from Australia, Igri, Barke and RGT Planet from Europe, and Morex and Hockett from North America) for the haplotypes of ten potential genes that may be associated with flowering time and responsiveness to photoperiod and light intensity (Methods S4). Among the ten genes, we identified five genes with a dominant haplotype in Australian varieties (Figure 1a). We revealed two distinctive haplotypes for *HvPhyC*. Clipper, Stirling, Morex, Hockett and RGT Planet share a haplotype (H1) characterized by an SNP mutation (G) in exon 1 and a 24 bp deletion in exon 4 (Figure 1a), and this haplotype is dominant in Australian varieties. For *HvCry1b*, Australian dominant haplotype H1 is characterized by a 7‐bp insertion in exon 1 and an SNP (T) in exon 2. All European or North American cultivars carry haplotype (H2) with a 7‐bp deletion and an SNP (G) (Figure 1a). A discriminant analysis revealed a proportion of Australian barley accessions having an overlapping genetic composition of *HvCry1b* not with European but with African barley (Methods S5, Figure S3), suggesting a possible non‐European origin of Australia's dominant haplotype in *HvCry1b*.

**Figure 1:**
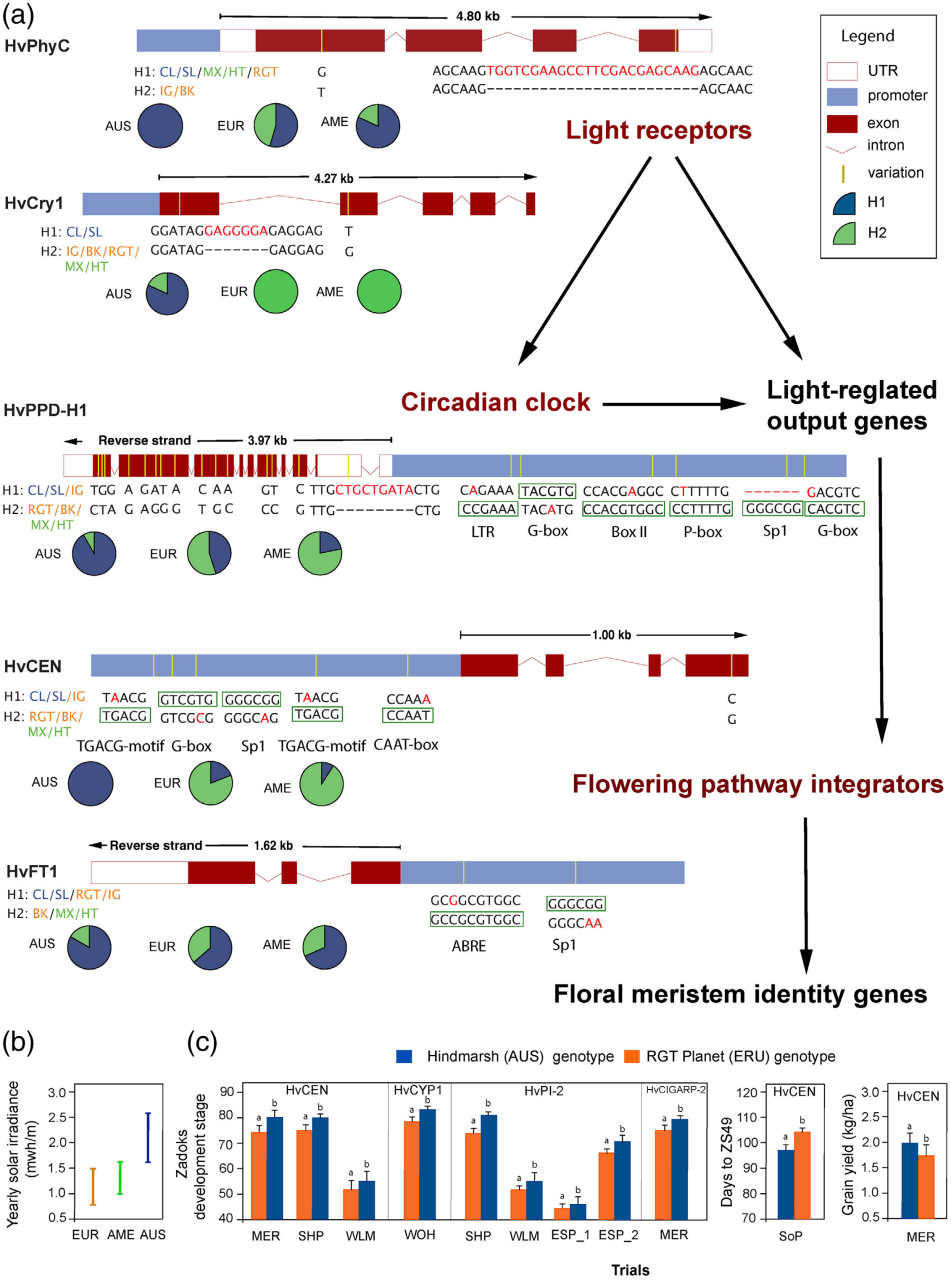
(a) Haplotypes of five critical genes associated with heading date and photoperiod sensitivity in barley from Australia, Europe, and North America. The light‐regulated flowering pathway followed Cao *et al*. (2021). The proportion of haplotypes was estimated by analysing 56 varieties (Table S12). Cis‐regulatory elements were determined using the webtool PlantCare (Lescot *et al*., 2002). (b) Yearly total solar irradiance in Australian, European and Australian major barley‐growing regions. (c) Phenotypic effect of genotype in Australian barley and European barley in recombination inbreeding line from crossing Australian barley variety Hindmarsh with European variety RGT Planet. Trail locations are given in Table S8. Lowercase letters indicate significant differences between genotypes (*P* < 0.05).

Australian barley production regions are exposed to stronger solar radiation than European and North American main barley‐growing regions (Figure 1b; Figure S4), which may have driven the significant differentiation in cryptochromes and phytochromes genes, such as *HvCry1b* and *HvPhyC*. For the *HvPPD‐H1* gene, Clipper, Stirling and European winter barley Igri share a haplotype with a 9‐bp insertion in the 5′UTR region and 14 SNPs. Apart from the distinctive variants identified in the coding region of *HvPPD‐H1*, we have identified haplotype‐specific variants in its promoter region. Changes in gene promoter regions and 5′UTR region could lead to differentiated phenotypes (Chen *et al*., 2022).

The gene *HvCEN* is a crucial regulator of flowering time in barley and has played an essential role in the agricultural expansion of barley cultivation. Clipper, Stirling and Igri share a haplotype (H1) with the previously reported SNP (G/C) in exon 4 encoding the amino acid Pro135 (HII‐Pro135, Comadran *et al*., 2012) that is most common among Australian cultivars (Figure 1a). The 135Ala type was found in the other four genomes. Haplotype‐specific variants are also present in the promoter region of *HvCEN* with possible functional implications on cis‐regulatory elements involved in light responsiveness and hormone response. Research suggests that the haplotype containing the Pro135 mutation in *HvCEN* is favoured in the European Mediterranean conditions because it confers early flowering to escape terminal drought (Fernández‐Calleja *et al*., 2021). Like the Mediterranean region, climate conditions in temperate Australia are also characterized by hot and dry summers. The 135Ala‐encoding haplotype is dominant in North American barley and those from a high latitude in Europe. Our results thus demonstrate the convergent selection of *HvCEN* in similar environments during the expansion from Europe to Australia and North America.

We verified the phenotypic effect of Australian genotypes of key phenology genes with a recombinant inbred lines population developed from crossing the Australian barley variety Hindmarsh® with the European variety RGT Planet^®^ (Methods S6). We generated a genetic map with the 270 recombinant inbred lines and evaluated their phenology and yield traits in six trials (Table S8). QTL mapping suggested that, for eight genetic markers, the Australian genotype (Hindmarsh) was associated with faster development or earlier flowering in at least one trial (*P* < 0.05, *t*‐test; Figure 1c). Four phenology genes, including *HvCEN*, were revealed to be within 0.5 cM of the eight genetic markers (Table S9). Australian genotype of the gene *HvCEN* was also associated with higher grain yield (*P* < 0.05, *t*‐test; Figure 1c).

Australian barley is photoperiod‐sensitive, which is beneficial in growth conditions that require barley to flower at a time with reliable rainfall, irrespective of the sowing date. In trials with different sowing times (30 days apart; Methods S7). Australian barley flowered at a relatively stable calendar date, irrespective of the sowing date, compared to the European varieties (Figure S5; Table S10). For Australian barley, the photoperiod sensitivity seemed to confer an advantage in environments with low and unpredictable rainfall in the sowing time (Figure S5d) and probable hot weather during maturation in the maturation stage (Figure S5e). We finally examined the influence on the earliness of heading date and photoperiod sensitivity of the haplotype of the five genes and observed differentiated phenotypes of haplotypes in two genes. Haplotype H1 (HII‐Pro135, Comadran *et al*., 2012) of *HvCEN*, the dominant haplotype in Australia, was associated with early flowering in five (out of seven) environments in the field trials (Figure S5; Table S11). Haplotype H1 of *HvPhyC*, the dominant haplotype in Australia, promoted early flowering time in late sowing by 5 days on average (Figure S5).

In summary, barley adaptation in the Australian environment involves selecting and subsequently enriching pre‐existing genetic variants within the European gene pool. Breeding activities have also introduced non‐European haplotypes. Selection for suitably adapted barley varieties in Australia has led to the fixation of several genes in flowering regulatory pathways. Australian varieties are dominated by one haplotype in each gene. Identifying these genes and haplotypes deepens our understanding of how breeding selections have shaped the genome architecture in Australian barley during its transition from Old World to New World.

## Funding

Funding was provided by the Grain Research and Development Corporation Australia. E.L.A. was supported by the Welch Foundation (Q‐1866), a McNair Medical Institute Scholar Award, an NIH Encyclopedia of DNA Elements Mapping Center Award (UM1HG009375), a US‐Israel Binational Science Foundation Award (2019276), the Behavioural Plasticity Research Institute (NSF DBI‐2021795), NSF Physics Frontiers Center Award (NSF PHY‐2019745), and an NIH CEGS (RM1HG011016‐01A1).

## Code availability

Codes used for analysis are available at https://github.com/lakeseafly/Australian_barley_genomes_MS_scripts.

## Supporting information


**Figure S1‐S6** Supplementary Figures.

**Table S1‐S14** Supplementary Tables.
**Method S1‐S7** Supplementary Methods.

## Data Availability

The Clipper and Stirling genome assembly, genome annotation and PacBio HiFi, RNA‐seq, Hi‐C sequence data are available at https://data.pawsey.org.au/public/?path=/wcga‐pangenome/Australian_barley_genomes_raw_data.
