# Supplementary material for: Genomic signatures of barley breeding for environmental adaptation to the new continents
Source: Plant Biotechnol J. 2023 Jul 27;21(9):1719–21. doi: 10.1111/pbi.14077 (PMC10440980; doi:10.1111/pbi.14077)
Supplement: Supplementary file 1 — Figure S1‐S6 Supplementary Figures. [file PBI-21-1719-s001.docx]

**Supplemental Data**

**Genomic signatures of barley breeding for environmental adaptation to the new continents**

Haifei Hu^1,2^, Penghao Wang^1^, Tefera Tolera Angessa^1^, Xiao-Qi Zhang^1^, Kenneth J. Chalmers^3^, Gaofeng Zhou^1^, Camilla Beate Hill^1^, Yong Jia^1^, Craig Simpson^4^, John Fuller^4^, Alka Saxena^5^, Hadi Al Shamaileh^5^, Munir Iqbal^5^, Brett Chapman^1^, Parwinder Kaur^6^, Olga Dudchenko^7,8^, Erez Lieberman Aiden^6,7,8,9,10^, Gabriel Keeble-Gagnere^11^, Sharon Westcott^12^, David Leah^13^, Josquin F Tibbits^11^, Robbie Waugh^4^, Peter Langridge^3^, Rajeev Varshney^1,14^, Tianhua He^1*^, Chengdao Li^1,12,14*^

^1^Western Crop Genetics Alliance, Centre for Crop & Food Innovation, Food Futures Institute, College of Science, Health, Engineering and Education, Murdoch University, WA Australia

^2^Rice Research Institute & Guangdong Key Laboratory of New Technology in Rice Breeding, Guangdong Academy of Agricultural Sciences, Tianhe, Guangzhou, 510640, China

^3^School of Agriculture, Food and Wine, University of Adelaide, Waite Campus, Glen Osmond, South Australia, Australia

^4^The James Hutton Institute, Dundee, UK

^3^School of Agriculture, Food and Wine, University of Adelaide, Waite Campus, Glen Osmond, South Australia, Australia

^4^The James Hutton Institute, Dundee, UK

^5^Genomics WA, University of Western Australia, Harry Perkins Institute of Medical Research and Telethon Kids Institute, QEII Campus, Nedlands, WA 6009, Australia

^6^School of Agriculture & Environment (SAgE), the University of Western Australia, Perth, WA 6009, Australia

^7^The Center for Genome Architecture, Department of Molecular and Human Genetics, Baylor College of Medicine, Houston, TX 77030, USA

^8^Center for Theoretical Biological Physics, Rice University, Houston, TX 77005, USA

^9^Shanghai Institute for Advanced Immunochemical Studies, ShanghaiTech, Pudong, China

^10^Broad Institute of MIT and Harvard, Cambridge, MA, USA

^11^Agriculture Victoria, Department of Jobs, Precincts and Regions, Agribio, 5 Ring Rd, La Trobe University, Bundoora, Victoria 3083 Australia

^12^Agriculture and Food, Department of Primary Industries and Regional Development, South Perth, WA, Australia

^13^Seed Force, VIC3630, Australia

^14^Centre for Crop & Food Innovation, State Agricultural Biotechnology Centre, Food Futures Institute, Murdoch University, WA Australia

***Corresponding Authors**

**Chengdao Li (c.li@murdoch.edu.au)**

**Tianhua He (Tianhua.He@murdoch.edu.au)**

**Supplementary Methods**

**Methods S1: Long reads sequencing, de novo assemble and gene annotation of the two Australian barley varieties "Clipper" and "Stirling"**

*Plant materials and DNA extraction for sequencing*

This study used Australian barley cultivars, Stirling and Clipper, for long-read sequencing and assembly. Clipper is one of the first modern barley varieties bred in Australia and was released in 1968 from one of the first full-time breeding programs at the Waite Agricultural Research Institute in South Australia. Stirling was bred in Western Australia and released in 1987. The seed resources were from the barley pedigree collection at the Department of Primary Industry and Regional Development, Western Australian Government. The seeds from a single plant were planted in pots with premium potting mix and grew in the new glasshouse of the Grain Research Hub on the Murdoch University campus. Three-week-old seedlings with four leaves were harvested for DNA extraction. We collected approximately four grams of each freshly harvested barley seedling that were snap-frozen in liquid nitrogen and ground to a fine powder in the presence of liquid nitrogen with a mortar and pestle, transferred to a 50 ml plastic tube, and kept in liquid nitrogen for use. We added 40 ml of lysis buffer containing ten mM Tris-HCl, pH 8.0, 20 mM EDTA, 500 mM Guanidine-HCl, 200 mM NaCl, 100 µg/ml of Pectinase and Cellulase, and 1% Triton® X-100 to the powder and mix by shaking and inverting until no lumps. We incubated the tube at 40°C for one hour with gentle agitation. Then we further added DNase -free RNase A to the lysis system at 50 µg/ml, and continued incubation for a half-hour at 40°C. Finally, we added Proteinase K to the 100 µg/ml tube and incubated for two more hours at 42°C. The tube was centrifuged at 4000 g in an Allegra X-15R centrifuge (Beckman Coulter) for 20 min at 20°C. We transferred the supernatant to an equilibrated Qiagen Genomic-tip 500/G. Protocols provided by Qiagen were followed for equilibration, washing and elution of the tip. The pure DNA collected from the Genomic-tip was precipitated with isopropanol, and the DNA coils were spooled out and washed with 70% ethanol. Air-dried DNA pellet was then dissolved in 100 µl TE buffer.

The final purified DNA was quantified on a Nanodrop 1000 and a Qubit® Fluorometer with dsDNA, Broad-Range assay. About 300 to 500 ng DNA was loaded on a 0.75% agarose gel and electrophoresis at 40 V for 5 to 6 hours and visualized on a Gel Doc on UV light. I µl of lambda DNA from Promega (411 ng/µl, 48.5 kb in size) was used as a reference. Before being sent to Genomics WA (Nedlands, Western Australia), the DNA samples were sent to the Genomics Lab on Murdoch University campus for further quality check with an Agilent Genomic DNA ScreenTape System. Both DNA samples isolated from barley cultivar Stirling and Clipper were shown good quality in purity and size from the above test measures.

*Long-read library preparation and sequencing*

HiFi SMRT bell libraries were prepared from each Barley sample using SMRT Bell express template preparation kit (Pacific BioSciences Catalog 100-938-900). The quality of DNA was checked using Nanodrop (Thermofisher Scientific), Qubit (Thermo Fisher Scientific) and Femto pulse (Agilent Technologies) before library preparation. In brief, libraries were prepared by shearing DNA using Diagenode Megaruptor-3 followed by fragment size check on Femto pulse. Fragmented DNA was damage repaired and end-repaired after removal of single-strand overhangs followed by SMRTbell adapter ligation, nuclease treatment and size selection on Blue Pippin prep. Library quality was checked on Femto Pulse and Qubit, before sequencing each sample on four 8M SMRT cells using Sequel II sequencing kit v2.0 (pacific BioSciences catalog 101-820-200) on PacBio Sequel II in CCS mode with a movie time of 30 hours each.

*Hi-C library preparation and sequencing*

*In situ* Hi-C was performed as described previously (Rao et al., 2014) using frozen leaves from Clipper and Stirling. Briefly, frozen leaf tissue was crosslinked, ground and then lysed with nuclei permeabilized. DNA was processed with MboI restriction enzyme and the overhangs were filled in by incorporating a biotinylated base. Free ends were then ligated together in situ. Crosslinks were reversed, the DNA was sheared to 300–500 bp and then biotinylated ligation junctions were recovered with streptavidin beads.

Standard Illumina library construction protocol was used for DNA sequencing for Clipper and Stirling. Briefly, DNA was end-repaired using a combination of T4 DNA polymerase, Escherichia coli DNA Pol I large fragment (Klenow polymerase), and T4 polynucleotide kinase. The blunt, phosphorylated ends were treated with Klenow fragment (3′ to 5′ exo minus) and dATP to yield a protruding 3- 'A' base for ligation of Illumina's adapters which have a single 'T' base overhang at the 3' end. After adapter ligation, DNA was PCR amplified with Illumina primers for 14 cycles and library fragments of 400–600 bp (insert plus adaptor and PCR primer sequences) were purified using SPRI beads. The purified DNA was captured on an Illumina flow cell for cluster generation. Libraries were sequenced on the NextSeq600 following the manufacturer's protocols. The resulting libraries were sequenced to yield approximately 25× coverage of the barley genome.

*RNA-Seq preparation and sequencing*

Three independent replications of five developmentally distinct barley tissues. Clipper and Stirling were sampled for RNA isolation and RNA-seq analysis (Table S13). These included: developmentally equivalent coleoptiles sampled 4-8 days post imbibition (including germinating embryos, mesocotyl and seminal roots); 14-20 day shoots (~5-6cm immediately above the germinated seed to the node of the first true leaf); roots from the same plantlets (all roots from below the germinated grain); 1cm inflorescences sampled 5-6 weeks post-germination; developing caryopses sampled ~15 days post anthesis. All samples were harvested and immediately immersed in liquid N and then stored at -80oC prior to RNA extraction. Total RNA extraction was done using the manufacturer's instructions using the Macherey-Nagel NucleoSpin RNA Plant, Mini kit. All tissues included the kit DNAse step. Seedling root and caryopsis tissues included a Plant Isolation aid (Invitrogen) (equal volume of Plant isolation aid/g of tissue) and centrifugation before RNA isolation. Total RNA was quality checked using a Bioanalyzer 2100 (Agilent), and RNA samples with the best RIN were used for RNA-seq library preparation. The Clipper Caryopsis rep3 sample was degraded and not sequenced. RNA for Illumina RNA-seq was aliquoted from the same sample. Illumina RNA-seq library preparation and RNA-seq were carried out by Novogene (HK) Company Limited, Hong Kong. The 30 libraries (3 bioreps x 5 tissues x 2 genotypes) were prepared using NEBNext® Ultra™ Directional RNA Library Prep Kit and sequenced using Illumina NovaSeq 6000 S4 (PE 150).

*Genome assembly and validation*

The clean PacBio HiFi reads were assembled using Hifiasm (v.0.15) (Cheng et al., 2021) with default parameters. Chromosome-length scaffolds were generated by aligning the raw HiC-reads to the draft assembly using Juicer (v.1.6) (Durand et al., 2016) with the resulting alignments processing by the 3D-DNA pipeline (v.19) (Dudchenko et al., 2017) to generate the candidate chromosome-length assemblies. These candidate assemblies were reviewed and curated using Juicebox Assembly Tools (v.1.11.08) (Robinson et al., 2018). To examine the genome assemblies, we compared our genomes with the barley assembly of Morex V3 using MCScanX (Wang et al., 2012). BUSCO V3 (Simao et al., 2015) with eukaryota_odb9 was used to assess the completeness of the assemblies. Centromeric repeats were identified using BLAST+ (version 2.2.3, E-value < 1e − 5) (Camacho et al., 2009) search barley centromere-specific [AGGGAG]^5^ repeats against the genome sequence.

*Gene annotation*

The *de novo* transposable element (TE) library was generated for the Clipper and Stirling genomes using EDTA v1 (Ou et al., 2019). The TE library was used to screen the genomes to identify repeat sequences. MAKER2 (Holt and Yandell, 2011) was used to annotate the Clipper and Stirling genomes. Three sets of evidence, including gene prediction evidence, transcript evidence and protein evidence, were provided as the annotation inputs. Gene evidence included *ab initio* gene prediction from SNAP (Korf, 2004) generated by MAKER2 annotation toolkit, Augustus (Stanke and Morgenstern, 2005) and Genemark (Tang et al., 2015) generated by BRAKER2 (Bruna et al., 2021) annotation pipeline. RNA-Seq data of five different tissues (coleoptile, shoot, root, developing inflorescence and caryopsis) were used for the transcript evidence. Adapter and low-quality sequences were removed from the raw RNA-seq reads using Trimmomatic (Bolger et al., 2014) v0.3.6 and mapped to the Clipper and Stirling genomes using HISAT2 (Kim et al., 2015). The resulting alignments were assembled to generate gene models using StringTie (Pertea et al., 2015). The protein sequences were downloaded from the barley pangenome project. The redundant sequences were removed to generate the representative barley protein sequences using cd-hit (Li and Godzik, 2006) v4.6.8 (-c 0.9). Protein evidence was generated by aligning the representative barley protein sequences using Exonerate (Slater and Birney, 2005) v.2.2. Finally, gene prediction, transcript and protein evidence were integrated for gene annotation by MAKER2. Genes were defined as high confidence genes using a similar method as Mascher et al. (2017), where if they had a significant BLAST hit to reference proteins and if their representative protein had a similarity to the respective template sequence above a threshold which we determined based on the origin of template sequences (> 60% for *Arabidopsis thaliana*, sorghum and rice, > 65 % for *Brachypodium distachyon*, and > 85 % for barley). Gene functional annotations were performed by SwissProt BLAST search (e-value<1e-10 and identity >50%) (Camacho et al., 2009).

**Methods S2 Population structure and phylogenetic relationships**

*Variant calling*

Three sets of data were used for variant calling, including 56 barley varieties with ~10× coverage of WGS, 325 varieties with 0.1-0.5x coverage of WGS and 325 varieties with target enrichment sequencing with ~20x coverage in targeted phenology gene regions. The 56 barley varieties were cultivated barley varieties grown in Europe, Australia and North America, and their sequences are publicly available in NCBI. The list of accessions, their geographic origin, NCBI Bioproject numbers are given in Table S12. The low coverage of WGS and target enrichment sequencing of the 325 cultivated barley with origin from Australia, Europe and North America were obtained from Hill et al. (2019a, 2021) (Table S11). The raw sequence data (.fastq files) are stored at ArrayExpress (https://www.ebi.ac.uk/arrayexpress/experiments/E-MTAB-7362/).

Clean reads of selected barley accessions were mapped to the Clipper reference genome using BWA MEM v0.7.17 (Li, 2013). Default settings were used and duplicates were removed by Picard tools (http://broadinstitute.github.io/picard/). Reads were realigned by GATK v3.8-1-0 RealignerTargetCreator and IndelRealigner(McKenna et al., 2010), followed by variant calling using GATK HaplotypeCaller. The resulting SNPs were filtered (QD < 2.0 || MQ < 40.0 || FS > 60.0 || QUAL < 60.0 || MQrankSum < −12.5 || Read- PosRankSum < −8.0) to remove low-quality SNPs. High-confidence SNPs were obtained by further filtering out the SNPs with minor allele frequency <0.05 and missing genotype rate > 10% using VCFtools (Danecek et al., 2011). The SNP-based phylogenetic trees of 56 barley accessions (Table S12) and 325 barley accessions (Table S11) were constructed using IQ-tree respectively (Nguyen et al., 2015) using a maximum likelihood method (with the parameters -alrt 1000 -bb 1000).

Nucleotide diversity values (π) were calculated using Pixy (Korunes and Samuk, 2021) using a 100-kb window. The genetic differentiation (Fst) between subpopulations was computed using a 100-kb sliding window and a step size of 10 kb with VCFtools (v0.1.15) (Danecek et al., 2011). XP-CLR (python version) (Chen et al., 2010) was used to calculate the composite likelihood and identify selected regions between the Australian and European barley populations and the American and European barley populations. The selected regions were detected using a step size of 10 kb and a 100-kb sliding window across each chromosome. The genomic regions with the top 1% XP-CLR scores above were considered under selection.

**Methods S3 Gene Presence/Absence variants analysis**

Sequencing reads of 325 varieties with target enrichment data were aligned to the Clipper genome using Bowtie2 (Langmead & Salzberg, 2012) v2.3.3.1 (–end-to-end –sensitive -I 0 -X 1000). We adopted the gene PAV detection method from Bayer et al.'s study (2022). A gene was considered as missing when the horizontal coverage across exons of the gene was <20% and the vertical coverage was <2×. To further validate the PAV detection accuracy, we manually examined the PAVs of three known phenology genes using IGV version 2.14.0 (Robinson et al., 2011) (Figure S6). We checked present and absent genes' read coverage in five selected Australian and European varieties. A PAV matrix was generated, showing the presence or absence of each gene for each accession. Statistical significance of PAV frequency changes between European and Australian barley populations and European and North American barley populations were determined by Z-score statistics. P-values were adjusted for multiple comparisons using the Bonferroni method. PAVs with an adjusted p-value < 0.05 were identified as under significant frequency changes between populations.

**Methods S4 Phenology genes, haplotypes, and their phenotypic effect**

Breeding activities in Australia have begun with selecting varieties with optimal phenology adapting to flexible sowing date that needs to maintain yield in transient and unpredictable variation in seasonal weather during the seeding season. We examined all the genes likely involved in conferring such optimal phenology in Australia's growing condition. Our previous study used targeted enrichment and field trials in seven environments and identified 122 genes associated with flowering time among 896 worldwide barley accessions (He et al., 2019; Hill et al., 2019, Table S14). Here we re-analyzed the dataset with only varieties from Australia, North America, and Europe included (325 varieties) (field trials for flowering time differentiation, Table S11). We identified the genes differentiating flowering time among Australia, North America, and European varieties. Consequently, six genes with significant SNPs located in either the gene coding regions or promoter regions based on previous SNP-based GWAS analysis (Hill et al., 2021) were identified to determine flowering time among barley from the three regions (*HvRLPK, HvSTK, HvPPd-H1, HvAG1, HvCEN, HvPhyC*). We also included genes annotated as flowering genes and were in high population differentiation regions (*HvCry1b*). Additional genes examined include those previously reported as related to flowering time and associated with heading date and responsiveness to photoperiod (*HvFT1, HvCO1, HvGA20ox2*) (Cao et al., 2021; Fernández-Calleja et al., 2021).

In total, we were able to analyze ten genes in detail. For each gene, we extracted their coding sequence and 5'UTR (if annotated) and 2,000 bp promoter sequence from the start site of the seven genomes (Clipper, Stirling, Morex V3, Hockett, Barker, RGT Planet, and Igri). Sequences were mapped to Clipper assembly, and variants in the exons and 5'UTR were detected, manually inspected, and recorded using CLC Genomic Workbench (QIAGEN, Aarhus, Denmark). Cis-regulatory elements in the 2,000 bp promoter sequences were searched using the web tool PlantCare (Lescot et al., 2002). Haplotypes were defined by sharing the same variants in the exons and 5'UTR, and the same difference in cis-regulatory elements. The haplotype that Clipper harboured was designated as H1. A machine learning-based tool PPVED was used to predict the effect of single amino acid substitution on the protein function of these genes (Gou et al., 2022).

Once the haplotypes were distinguished from the seven assemblies, we searched the 56 varieties with whole-genome sequencing for their corresponding haplotype. We also designated haplotypes for the 325 varieties that we investigated their flowering time in seven different environments. We genotyped their variation in putative flowering genes through targeted enrichment (Hill et al., 2019a, b), where we were able to define the haplotype of >80% of varieties depending on the environment and gene. Once their haplotypes were determined, we calculated and compared the phenotypic effect (flowering time, as measured by days to ZS49) of each haplotype for each gene. Note that there was no population differentiation between the group with the H1 haplotype and the one with the H2 haplotype.

**Methods S5 Discriminant analysis of the possible African origin of *HvCry1b* gene haplotype**

Discriminant analysis was used to explore the possible African origin of the *HvCry1b* gene haplotype. Discriminant analysis distributes samples into categories of the same type. Hill et al. (2019) sequenced *HvCry1b* gene in 25 barley accessions from Africa, 210 accessions from Australia, and 283 from Europe, and discovered the SNPs and small InDels in the samples using the target-capture method. We retrieved the 47 SNPs in the *HvCry1b* gene and chromosome region within 10 Mb upstream and downstream of this gene from Hill et al. (2019). For discriminant analysis, we interpreted SNPs as co-dominant with two alleles and assigned 1, 2, 3, or 4 to A, C, G, or T for each allele. We categorized the 518 samples into three groups according to their geographic origin. As we had far fewer samples from Africa than two groups, we did not attempt to assign each individual sample to a geographic group; instead, we detected whether some Australian samples were clustered with the African group rather than with the European group. Discriminant analysis was implemented with PAST v4 (Hammer et al. 2010).

**Methods S6 Field trials for photoperiod sensitivity of Australian, European, and North American barley**

Photoperiod sensitivity has been proposed as a critical adaptation to the growing condition in Australian barley. We investigated the differentiated phenotypic effect of different haplotypes of key genes. In the field trial in 2017 in South Perth, Western Australia, we sowed 180 barley varieties (including 66 Australian varieties, 41 European and 73 North American, all were genotyped at the putative flowering genes in Hill et al. 2019a, b) on 19 April and a month later on 19 May 2017 (Table S10). Each sample included at least five plants, and their development stage (days to ZS49, Zadoks et al. 1974) was recorded as equivalent to flowering time (Alqudah and Schnurbusch, 2017). Management of the field trial followed the standard practice in the region. The photoperiod sensitivity was measured by the days between the days to ZS49 on two sowing dates. Fewer days between the days to ZS49 on two sowing dates suggest more photoperiod sensitivity for a variety. Differentiation of flowering time between Australian, European and North American barley was evaluated with a one-way ANOVA test using SPSS v25 (Armonk, NY: IBM Corp). Statistical significance was taken at p <0.05.

**Methods S7 RGT Plant X Hindmarsh recombinant inbred lines and QTL mapping for functional validation of Australian dominant haplotypes**

*Recombinant inbred lines development and construction of the genetic map*

A cross was made at Murdoch University, Australia, between RGT Planet as a maternal plant and Hindmarsh as the pollen parent to develop the RGT Planet/ Hindmarsh lines. A BC1F1 was developed from a cross made between the F1 maternal plant RGT Planet/Hindmarsh and pollens from Hindmarsh to develop RGT Planet/Hindmarsh//Hindmarsh population. Single seed descent (SSD) and speed breeding approach were used to fast track the development of 233 fixed lines from the RGT Planet/Hindmarsh population and 37 lines from the RGT Planet/ Hindmarsh//Hindmarsh population to F6 generation that was used to construct the genetic map. Genomic DNA was extracted from the leaves of a single plant per line using the cetyl-trimethyl-ammonium bromide (CTAB) method (Edwards et al. 1991). Genotyping by sequencing was implemented using the DArTseq platform (DArT PL, Canberra, NSW, Australia). Sequences of flanking polymorphism detected by DArTseq were then aligned against the MorexV2 genome assembly to determine their physical positions.

*Evaluation of phenology traits*

The 270 lines derived from RGT Planet and Hindmarsh crosses were evaluated along with the two parental varieties, and 12 control varieties in six different environments in Western Australia (WA) and Victoria (VIC), Australia, during the 2019 growing season (Table S10). The entries were planted in partially replicated yield plots in regional locations in WA and harvested from plots with dimensions of 4 meters length by 1.1 meters width or 4 meters length by 1.7 meters width. The entries were planted in hill plots at South Perth, WA and planted in partially replicated one-meter-long row plots at Shepperton, VIC.

Days to first awn pipping (ZS49) were recorded in the South Perth environment by visiting the trial 2-3 times per week once the flowering had started. At all other sites, Zadok's growth stage was recorded on a specific date as a proxy phenology trait observed in each plot. Yield data in each trail was also obtained after the harvest. The data were subjected to ASReml Statistical Software (VSN International. 2021), Best Linear Unbiased Estimated (BLUE), and Best Linear Unbiased Predicted (BLUP) means were calculated to accommodate for the spatial effect in each environment.

*QTL mapping*

QTL mapping was implemented with MapQTL_6 (Van Ooijen, 2009) using the genotypic data, phenotypic data, and genetic map with the following parameters: grouping LOD criteria LOD=3, population types RIL at generation 4, number of lines 224, No mapping size threshold 2, No mapping distance threshold 15 cM, No mapping missing threshold 25%, and genetic mapping function kosambi (Table S15). Interval mapping was conducted for each trait from each trial, and the markers with a LOD value of > 3.0 were selected as cofactors. MQM mapping was executed to re-calculate the QTL. If the marker with the highest LOD value was inconsistent with the cofactor marker, the new marker was designated as cofactors and QTL was re-calculated. The gene is determined as the candidate gene if a known phenology gene is situated within 0.5 cM from the significant genetic marker. Phenotypic comparison Australian genotype and the European genotype was conducted with the Student's t-test using SPSS v25 (Armonk, NY: IBM Corp). Statistical significance was taken at p <0.05.

**References**

Alqudah, A.M. and Schnurbusch, T. (2017). Heading date is not flowering time in spring barley. Frontier in Plant Science 8: 896

Bayer, P.E., Valliyodan, B., Hu, H., Marsh, J.I., Yuan, Y., Vuong, T.D., Patil, G., Song, Q., Batley, J., Varshney, R.K., Lam, H.M., Edwards, D. and Nguyen, H.T. (2022) Sequencing the USDA core soybean collection reveals gene loss during domestication and breeding. *Plant Genome* **15**, e20109.

Bolger, A.M., Lohse, M. and Usadel, B. (2014) Trimmomatic: a flexible trimmer for Illumina sequence data. Bioinformatics 30: 2114-2120.

Bruna, T., Hoff, K.J., Lomsadze, A., Stanke, M. and Borodovsky, M. (2021) BRAKER2: automatic eukaryotic genome annotation with GeneMark-EP+ and AUGUSTUS supported by a protein database. NAR Genom Bioinform 3: lqaa108.

Cao, S., Luo, X., Xu, D., Tian, X., Song, J., Xia, X., Chu, C. and He, Z. (2021) Genetic architecture underlying light and temperature mediated flowering in Arabidopsis, rice, and temperate cereals. New Phytologist 230: 1731-1745.

Chen, H., Patterson, N. and Reich, D. (2010) Population differentiation as a test for selective sweeps. Genome Research 20: 393-402.

Cheng, H., Concepcion, G.T., Feng, X., Zhang, H. and Li, H., 2021. Haplotype-resolved de novo assembly using phased assembly graphs with hifiasm. Nature methods, 18(2), pp.170-175.

Camacho, C., Coulouris, G., Avagyan, V., Ma, N., Papadopoulos, J., Bealer, K. and Madden, T.L., 2009. BLAST+: architecture and applications. BMC bioinformatics, 10, pp.1-9.

Danecek, P., Auton, A., Abecasis, G., Albers, C.A., Banks, E., DePristo, M.A., Handsaker, R.E., Lunter, G., Marth, G.T., Sherry, S.T., et al. (2011). The variant call format and VCFtools. Bioinformatics 27: 2156-2158.

Dudchenko, O., Batra, S.S., Omer, A.D., Nyquist, S.K., Hoeger, M., Durand, N.C., Shamim, M.S., Machol, I., Lander, E.S. and Aiden, A.P. (2017). De novo assembly of the Aedes aegypti genome using Hi-C yields chromosome-length scaffolds. Science 356: 92-95.

Durand, N.C., Shamim, M.S., Machol, I., Rao, S.S., Huntley, M.H., Lander, E.S. and Aiden, E.L. (2016). Juicer Provides a One-Click System for Analyzing Loop-Resolution Hi-C Experiments. Cell System 3: 95-98.

Edwards, K., Johnstone, C., and Thompson, C. (1991). A Simple and Rapid Method for the Preparation of Plant Genomic DNA for Pcr Analysis. Nucleic Acids Research 19: 1349-1349.

Fernández-Calleja, M., Casas, A.M. and Igartua, E. (2021). Significant flowering time genes of barley: allelic diversity, effects, and comparison with wheat. Theoretical and Applied Genetics 134:1867-97.

Gou, X., Feng, X., Shi, H., Guo, T., Xie, R., Liu, Y., Wang, Q., Li, H., Yang, B., Chen, L. and Lu, Y. (2022). PPVED: A machine learning tool for predicting the effect of single amino acid substitution on protein function in plants. Plant Biotechnology Journal. https://doi.org/10.1111/pbi.13823

Hammer, Ø., Harper, D.A. and Ryan, P.D. (2001). PAST: paleontological statistics software package for education and data analysis. Palaeontologia Electronica 4, 9.

He, T., Hill, C.B., Angessa, T.T., Zhang, X.Q., Chen, K., Moody, D., Telfer, P., Westcott, S. and Li, C. (2019). Gene-set association and epistatic analyses reveal complex gene interaction networks affecting flowering time in a worldwide barley collection. Journal of Experimental Botany 70: 5603-5616.

Hill, C.B., Angessa, T.T., McFawn, L.A., Wong, D., Tibbits, J., Zhang, X.Q., Forrest, K., Moody, D., Telfer, P., Westcott, S., et al. (2019a). Hybridization-based target enrichment of phenology genes to dissect the genetic basis of yield and adaptation in barley. Plant Biotechnology Journal 17: 932-944.

Hill, C.B., Wong, D., Tibbits, J., Forrest, K., Hayden, M., Zhang, X.Q., Westcott, S., Angessa, T.T. and Li, C. (2019b) Targeted enrichment by solution-based hybrid capture to identify genetic sequence variants in barley. Sci Data 6, 12.

Hill, C.B., Angessa, T.T., Zhang, X.Q., Chen, K., Zhou, G., Tan, C., Wang, P., Westcott, S. and Li, C. (2021) A global barley panel revealing genomic signatures of breeding in modern Australian cultivars. The Plant Journal, 106, 419-434.

Holt, C. and Yandell, M. (2011) MAKER2: an annotation pipeline and genome-database management tool for second-generation genome projects. BMC Bioinformatics 12, 491.

Kim, D., Langmead, B. and Salzberg, S.L. (2015) HISAT: a fast spliced aligner with low memory requirements. Nat Methods 12, 357-360.

Korf, I. (2004) Gene finding in novel genomes. BMC Bioinformatics 5, 59.

Korunes, K.L. and Samuk, K. (2021) pixy: Unbiased estimation of nucleotide diversity and divergence in the presence of missing data. Mol Ecol Resour 21, 1359-1368.

Langmead, B. and Salzberg, S.L., 2012. Fast gapped-read alignment with Bowtie 2. Nature methods, 9(4), pp.357-359.

Lescot M, Déhais P, Thijs G, Marchal K, Moreau Y, Van de Peer Y, Rouzé P, Rombauts S. (2002). PlantCARE, a database of plant cis-acting regulatory elements and a portal to tools for in silico analysis of promoter sequences. Nucleic Acids Research. 30: 325-7.

Li, H. (2013) Aligning sequence reads, clone sequences and assembly contigs with BWA-MEM. arXiv preprint arXiv:1303.3997.

Li, W. and Godzik, A. (2006) Cd-hit: a fast program for clustering and comparing large sets of protein or nucleotide sequences. Bioinformatics 22, 1658-1659.

Mascher, M., Gundlach, H., Himmelbach, A., Beier, S., Twardziok, S.O., Wicker, T., Radchuk, V., Dockter, C., Hedley, P.E., Russell, J., et al. (2017). A chromosome conformation capture ordered sequence of the barley genome. Nature 544: 427-433.

McKenna, A., Hanna, M., Banks, E., Sivachenko, A., Cibulskis, K., Kernytsky, A., Garimella, K., Altshuler, D., Gabriel, S., Daly, M. and DePristo, M.A. (2010). The Genome Analysis Toolkit: a MapReduce framework for analyzing next-generation DNA sequencing data. Genome Research 20: 1297-1303.

Nguyen, L.T., Schmidt, H.A., von Haeseler, A. and Minh, B.Q. (2015). IQ-TREE: a fast and effective stochastic algorithm for estimating maximum-likelihood phylogenies. Molecular Biology and Evolution 32: 268-274.

Ou, S., Su, W., Liao, Y., Chougule, K., Agda, J.R.A., Hellinga, A.J., Lugo, C.S.B., Elliott, T.A., Ware, D., Peterson, T., et al. (2019). Benchmarking transposable element annotation methods for creation of a streamlined, comprehensive pipeline. Genome Biology 20: 275.

Pertea, M., Pertea, G.M., Antonescu, C.M., Chang, T.C., Mendell, J.T. and Salzberg, S.L. (2015). StringTie enables improved reconstruction of a transcriptome from RNA-seq reads. Nature Biotechnology 33: 290-295.

Rao SSP, Huntley MH, Durand NC, Stamenova EK, Bochkov ID, Robinson JT, Sanborn AL, Machol I, Omer AD, Lander ES, et al. 2014. A 3D map of the human genome at kilobase resolution reveals principles of chromatin looping. Cell 159: 1665–1680.

Robinson, J.T., Thorvaldsdóttir, H., Winckler, W., Guttman, M., Lander, E.S., Getz, G. and Mesirov, J.P., 2011. Integrative genomics viewer. Nature biotechnology, 29(1), pp.24-26.

Robinson, J.T., Turner, D., Durand, N.C., Thorvaldsdottir, H., Mesirov, J.P. and Aiden, E.L. (2018). Juicebox.js Provides a Cloud-Based Visualization System for Hi-C Data. Cell System 6: 256-258 e251.

Simao, F.A., Waterhouse, R.M., Ioannidis, P., Kriventseva, E.V. and Zdobnov, E.M. (2015) BUSCO: assessing genome assembly and annotation completeness with single-copy orthologs. Bioinformatics 31, 3210-3212.

Slater, G.S. and Birney, E. (2005) Automated generation of heuristics for biological sequence comparison. BMC Bioinformatics 6, 31.

Stanke, M. and Morgenstern, B. (2005) AUGUSTUS: a web server for gene prediction in eukaryotes that allows user-defined constraints. Nucleic Acids Research 33: W465-W467.

Tang S, Lomsadze A, Borodovsky M. 2015. Identification of protein-coding regions in RNA transcripts. Nucleic Acids Res 43: e78.

Van Ooijen JW. MapQTL 6, Software for the mapping of quantitative trait loci in experimental populations of diploid species. Kyazma BV; Wageningen, Netherlands: 2009

Wang, Y., Tang, H., Debarry, J.D., Tan, X., Li, J., Wang, X., Lee, T.H., Jin, H., Marler, B., Guo, H., Kissinger, J.C. and Paterson, A.H. (2012). MCScanX: a toolkit for detection and evolutionary analysis of gene synteny and collinearity. Nucleic Acids Res 40: e49.

Zadoks, J.C., Chang, T.T. and Konzak, C.F. (1974). A decimal code for the growth stages of cereals. Weed Research 14: 415-421.

**Supplementary Figures**

Figure S1: De novo genome assemblies of Australian barley varieties (Clipper and Stirling). A) Chromosome length of the Clipper and Stirling genomes. Outer (a) to inner (f) tracks: a, gene density b, centromere distribution c, Gypsy TE density d, Copia TE density e, DNA retrotransposon and f, syntenic relationships between the Clipper and Stirling genomes. Chromosome contact maps across the seven chromosome-length scaffolds of the Clipper (B) and Stirling (C) assemblies

Figure S2: Genomic diversity and differentiation of Australian (AUS), European (EUR), and North American (AME) barley populations. A) Phylogenetic relationships of the 56 barley cultivars from Australia, Europe, and North America show the European origin of Australian and North American barley. B) Statistics for genomic diversity and population differentiation between the Australian, European and North American barley. The numbers correspond to nucleotide diversity (π) in the different populations. C, D) Genome‐wide selected sweeps in EUR compared to AUS (C) and EUR compared to AME barley genomes (D). The red horizontal dashed lines indicate the cut-offs of the top 1% of XPCLR scores. Known phenology genes or homologous genes functionally characterised in rice that overlap with selected regions are annotated above the selection signals. E). There is a probability of significant change in the frequency of gene PAV in Australian barley compared to European varieties. F). Probability of significant change in frequency of presence of each PAV in North American barley compared to European varieties. Significance was taken at p < 0.05 with Bonferroni correction, which indicated the orange dashed line in both E and F.

Figure S3: Discrimination analysis of genetic variation of the gene *HvCry1b* within the Australian, European and African barley. The SNPs within the gene coding region and 1 Mb of upstream and downstream of the gene were used for discrimination analysis.


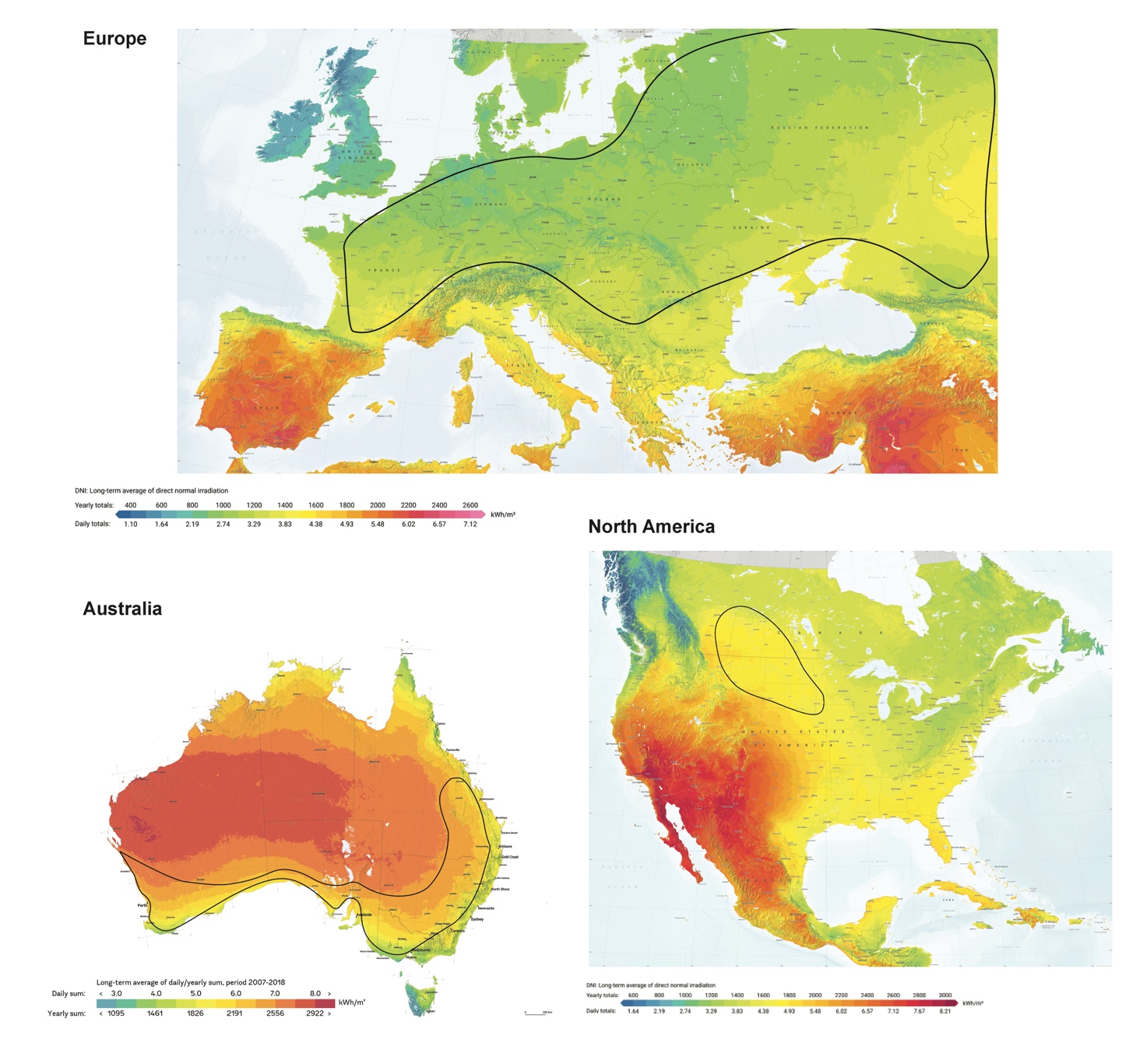


Figure S4: Solar irradiance in European, Australian and North American major barley production regions (encircled line). Map of solar irradiance was obtained SOLARGIS (<https://solargis.com>). European barley production region includes countries with barley production greater than 1 million tonnes (<https://www.fao.org/faostat/en/#search/barley>). Australian barley production region was obtained from He et al. (2022).


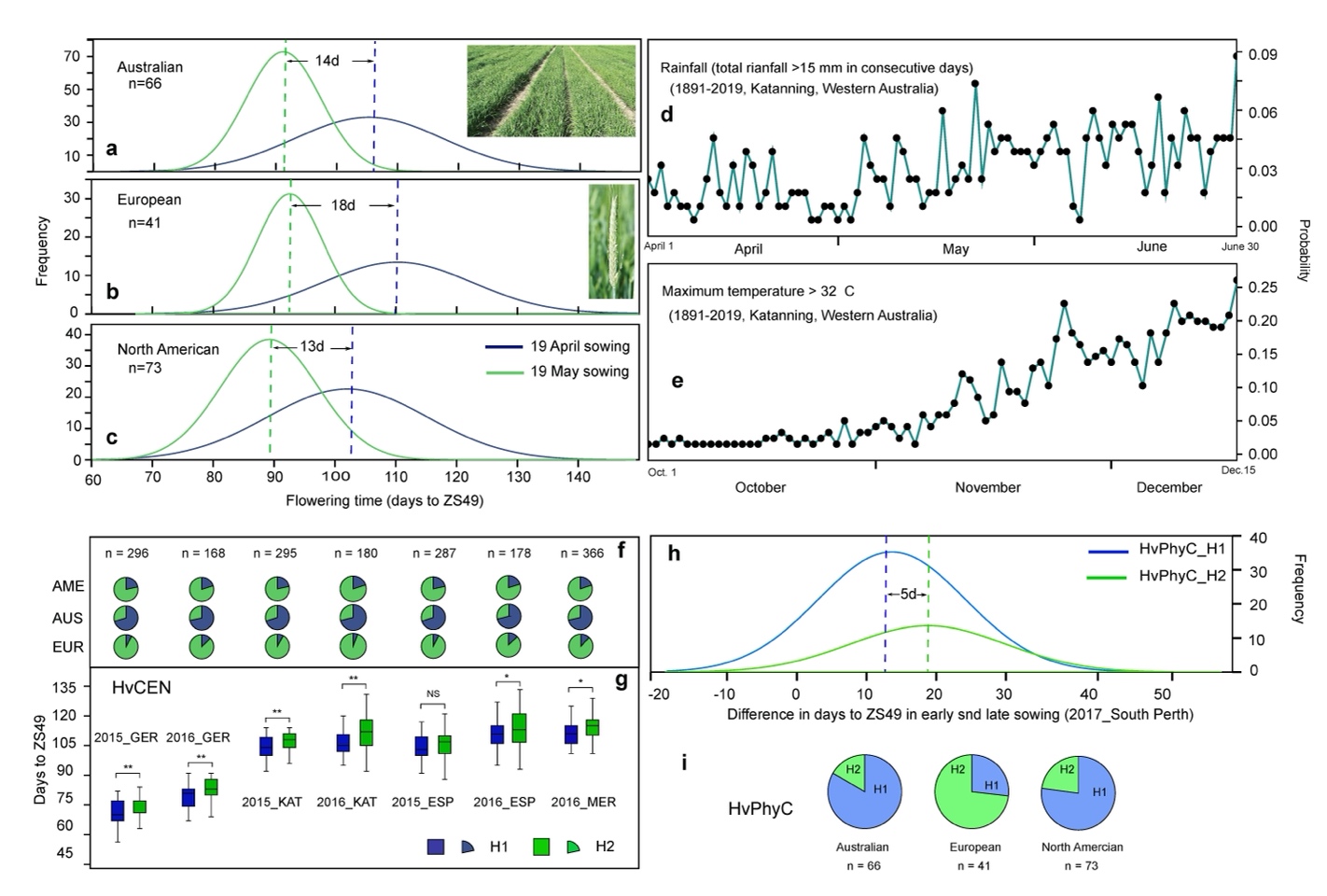


Figure S5: Genes and haplotypes associated with early heading and photoperiod sensitiveness in Australian barley and the determining climate in Australian barley growing condition. Frequency distribution of flowering time (days to ZS49) with response to different sowing dates (30 days apart) in Australian (A), European (B) and North American (C) barley. D) Rainfall reliability (probability with > 15 mmm rain within three consecutive days) in late autumn and early winter over 128 years recorded in Katanning, a typical barley production region in Western Australia. E) Probability of hot weather with maximum temperature > 32 ^o^C recorded in Katanning. F) The frequency distribution of the two haplotypes of *HvCEN* in Australian, European and North American barley samples in seven field trials. G) Phenotypic differentiation of the two haplotypes of *HvCEN* of different trials across the Western Australian Wheatbelt (GER: Geraldton, MER: Merredin, KAT: Katanning, and ESP: Esperance). *: p< 0.05, **: p<0.01; NS: non-significant. H) The differentiated effect of the two haplotypes in *HvPhyC* on the photoperiod sensitivity (ANOVA p < 0.05). I) The frequency distribution of the two haplotypes of *HvPhyC* in Australian, European, and North American barley samples in the trial with different sowing dates (30 days apart). Climate data were obtained from the Australian Bureau of Meteorology.

Figure S6: Manually checking the gene presence and absence of genes *HvSOC1*, *HvBM16* and *HvCO18* in Australian (AUS) barley and European (ERU) barley.

**Supplementary Tables**

Table S1: Assembly metrics and quality comparison of Clipper, Stirling and Morex V3 (Mascher et al. 2021)

Table S2: Average FST value of different chromosomes between AME and EUR populations and AUS and EUR populations.

Table S3: Selected regions between AUS and EUR.

Table S4: Selected regions between AME and EUR.

Table S5: Genes in the AUS vs EUR selected regions.

Table S6: Genes in the AME vs EUR selected regions.

Table S7: Genes PAV with significant presence frequency change when compared the Australian barely with European barley varieties.

Table S8: Details of Hindmarsh and RGT Planet recombinant inbred lines trials

Table S9: QTL mapping Hindmarsh and RGT Planet recombinant inbred lines

Table S10 Flowering time of 2017 trial in South Perth WA under different sowing times of Australian, European, and North American barley

Table S11: Barley accessions from Australia, Europe and North America, and their flowering time from 2015-2017 were recorded in seven environments for analysis (obtained from Hill et al., 2019b)

Table S12: Meta information of the 56 barley cultivars with deep sequencing

Table S13: Metadata of RNA-seq of Clipper and Stirling

Table S14: The significant GWAS signals of flowering times and the closest gene within 200kb up and downstream of the GWAS signals from previous SNP-based GWAS analysis (Hill et al., 2021)
